# Could the DSM-5 Cultural Formulation Interview Hold Therapeutic Potential? Suggestions for Further Exploration and Adaptation Within a Framework of Therapeutic Assessment

**DOI:** 10.1007/s11013-021-09761-2

**Published:** 2021-12-08

**Authors:** Mattias Strand, Sofie Bäärnhielm

**Affiliations:** 1 Transcultural Centre, Stockholm Health Care Services, Region Stockholm, Solnavägen 4, 113 65 Stockholm, Sweden; 2 Centre for Psychiatry Research, Department of Clinical Neuroscience, Karolinska Institutet & Stockholm Health Care Services, Region Stockholm, 171 77 Stockholm, Sweden

**Keywords:** Therapeutic assessment, Cultural formulation, Patient-centered care, Patient participation, Patient involvement

## Abstract

The Cultural Formulation Interview (CFI), included in the fifth edition of the *Diagnostic and Statistical Manual of Mental Disorders*, is a person-centered instrument for systematically appraising the impact of cultural factors in psychiatric assessment. A number of key areas in the future development of the CFI have been identified in order to ensure further clinical uptake. In this paper, we suggest that applying a Therapeutic Assessment (TA) approach in using the CFI—i.e., framing the interview in a way that gives primacy to its self-transformative potential by explicitly focusing on those issues that are seen as the most urgent, relevant, and meaningful by the patient—could prove helpful in alleviating patients’ suffering beyond what is achieved by merely collecting relevant cultural information that may inform diagnosis and subsequent treatment interventions. The TA methodology has been designed as a collaborative approach to psychological assessment in which the assessment procedure itself is meant to induce therapeutic change. This is achieved by explicitly focusing on the particular questions and queries that patients have about themselves with respect to their mental health problems or psychosocial well-being; these questions are then allowed to guide the assessment process and the interpretation of the findings. We suggest a number of potential modifications to the related Outline for Cultural Formulation and to the CFI content that could strengthen a TA-inspired focus. With this paper, we do not claim to offer a definitive integration of the TA approach in using the CFI but hope to further the discussion of a therapeutic potential of the instrument.

## Introduction: The Cultural Formulation Interview in Current Practice

Cultural differences in how patients and clinicians conceptualize health, illness, and treatment can create barriers in terms of help seeking, patient–clinician communication and assessment, making diagnoses, engaging patients in treatment, and evaluating outcomes. Differences in gender, age, ethnicity, socioeconomic background, educational level, language, sexual orientation, religious beliefs, occupation, and/or disability, etc. may contribute to misunderstandings, lack of insight by clinicians into the patients’ contexts, and failure to establish rapport and trust. Failing to recognize and address such barriers can ultimately result in poor treatment adherence, negative outcomes, and prolonged illness.

In order to help clinicians address these issues in a systematic manner, the Outline for Cultural Formulation (OCF) was introduced in the fourth edition of the *Diagnostic and Statistical Manual of Mental Disorders* (DSM-IV) (American Psychiatric Association, 1994), published in 1994. The OCF offered clinicians in psychiatry a conceptual framework for identifying and addressing cultural impact on patient experiences of illness and care during clinical assessment. Building on an updated version of the OCF (Aggarwal, 2013; Lewis-Fernández et al., 2014), the Cultural Formulation Interview (CFI) was developed and included in the fifth edition of the *Diagnostic and Statistical Manual of Mental Disorders* (DSM-5) (American Psychiatric Association, 2013), published in 2013. The core component of the CFI includes 16 open-ended questions on topics related to patients’ cultural understanding of health and illness; cultural and social context, stressors, and support; the role of cultural identity in coping and help seeking and the patient–clinician relationship. In addition, there are 12 supplementary modules that can be used to expand on the basic assessment and to address topics relevant for specific populations (such as children and adolescents, the elderly, or migrants and refugees).

In the DSM-5 field trials, the CFI was found to be a feasible, acceptable, and clinically useful tool for the assessment of relevant cultural factors (Lewis-Fernández et al., 2017). Not least, the CFI has been shown to enhance patient rapport through increased satisfaction associated with the person centeredness of the clinical interview (Aggarwal et al., 2015). Implementation and utilization of the CFI and related interviews based on the OCF in various settings and contexts have been reported (Bäärnhielm, 2012; Bäärnhielm and Scarpinati Rosso, 2009; Fortuna, Porche, and Alegría, 2009; Groen et al., 2017; Paralikar, Deshmukh and Weiss, 2020; Paralikar et al., 2015; Scarpinati Rosso and Bäärnhielm, 2012; Skammeritz et al., 2020; Wallin, Dahlin, Nevonen, and Bäärnhielm, 2020). The CFI has also been utilized in child and adolescent psychiatry (La Roche and Bloom, 2018; Rousseau et al., 2018). Patients’ family members and relatives have generally found the CFI to be useful and acceptable (Hinton et al., 2015). Preferred models for clinician training in utilizing the CFI have been analyzed (Aggarwal et al., 2016) and a fidelity instrument for the assessment of manualized CFI use has been developed (Aggarwal et al., 2014). (It should be noted that some of the studies referenced here used the DSM-5 field study beta version of the CFI, which differs slightly from the final version published in the DSM-5.)

Nevertheless, a number of barriers to optimal implementation and utilization of the CFI have also been noted (Aggarwal et al., 2013). Issues raised by patients in the DSM-5 field study (i.e., responding to the beta version of the CFI) include redundancy due to perceived similarities between the CFI and standard clinical interviews, unwillingness to discuss the past or religious matters, and difficulties in understanding certain questions. Issues raised by clinicians in the DSM-5 field study include potential lack of purpose and relevance—if busy clinicians do not believe that the CFI adds important information, they may simply not use it—and difficulties in finding the time to perform the interview in its entirety. In response to these comments, some adjustments were made to the CFI before its final publication in the DSM-5 (Aggarwal et al., 2013). In child and adolescent psychiatry, it has been suggested that a separate supplementary module for the youngest children needs to be developed, integrating the CFI with developmentally appropriate assessment tools such as play therapy (La Roche and Bloom, 2018). Opinions also differ regarding the practical use of the CFI in adults. Whereas published case studies have often emphasized broader social and cultural context, some authors hold that in everyday use in a medical setting, the interview should remain tightly focused on clinically relevant details; indeed, this was one of the main reasons behind the development of the CFI from the previous OCF (Caballero Martínez, 2009; Mezzich et al., 2009).

In a recent editorial piece by Lewis-Fernández, Aggarwal, and Kirmayer (2020), three key areas in the future development of the CFI are highlighted. First, the role of the CFI in potentially transforming how clinicians conceive of standard clinical assessment in psychiatry is raised, emphasizing the centrality of interpersonal, social, and cultural context in all psychiatric interviews. In order to allow this perspective to guide the diagnostic process and to signal to patients that their experiences and views are important, it is recommended that the CFI precedes the standard symptom-focused interview whenever possible. Second, the dissemination and implementation of the CFI need to be strengthened (Lewis-Fernández et al., 2020). This includes optimizing the use of CFI in different clinical settings, incorporating CFI information into treatment plans, and improving training. The usefulness of the CFI is clearly evident; yet, the impression that it has not reached its full potential in terms of implementation in healthcare services appears to be widespread in the field. In order to further justify the use of the CFI and make policymakers and service providers more likely to implement it properly, analyzing the cost effectiveness of the instrument through health economic evaluations may be helpful. Third, it is suggested that there is room for improvement of the CFI instrument from several perspectives (Lewis-Fernández et al., 2020). For example, the CFI questions on cultural identity remain somewhat difficult to understand for some patients and clinicians. A stronger emphasis on social determinants of health, including availability of and access to various types of resources, has also been suggested in order to improve the assessment procedure (Paralikar et al., 2020; Weiss et al., 2021). Moreover, there may be various ways to structure the information that is collected through the use of the CFI (Kirmayer, 2015) and complementary methods for eliciting cultural information, such as timelines, drawings, or maps, could be explored (Arnault and Shimabukuro, 2011). The creation of an additional module for the assessment of issues related to bereavement has also been suggested (Smid et al., 2018).

While we fully agree with these suggestions, we would like to suggest a fourth potential avenue for the CFI, namely that it could be useful not only as an assessment tool but also additionally as a therapeutic tool in its own right. Surely, it is often emphasized that the CFI can be useful in improving treatment outcomes; however, this usefulness generally hinges on aiding the clinician in gathering information on the cultural context of the patient, making a correct culturally informed diagnosis, exploring patients’ help-seeking habits, and improving trust and treatment adherence. As a complement to this perspective, we want to discuss the literature on so-called *Therapeutic Assessment* (TA)—an approach where the assessment procedure itself is designed to induce change in the patient—and compare it to conceptual features of the CFI. Our objective is to demonstrate that the use of an explorative instrument such as the CFI, when applied in a way that gives primacy to its self-transformative potential by explicitly focusing on those issues that are seen as the most urgent, relevant, and meaningful by the patient, could prove helpful in alleviating patients’ suffering beyond what may be achieved by mainly collecting relevant cultural information and allowing it to inform subsequent treatment interventions. Thus, we argue that in addition to informing clinical decision making related to diagnosis and treatment, the CFI could hold a therapeutic potential in and by itself.

## Clinical Assessment as a Therapeutic Process

In the history of mental healthcare, psychological assessment has mostly been thought of as a tool in diagnosing psychiatric disorders, facilitating communication between clinicians, and planning treatment interventions (Finn and Tonsager, 1997). Since the middle of the twentieth century, however, psychologists have experimented with using assessment instruments in ways that may also promote therapeutic change. These models grew out of the humanistic movement that saw conventional use of testing as a reductionist and questionable practice (Fischer, 1985). The TA approach was developed by Stephen Finn and colleagues at the Centre for Therapeutic Assessment in Austin, Texas, from the late 1980s and onwards. The model is built around the idea that assessment feedback is more readily accepted by patients if it in some way relates to what they already know and think about themselves—i.e., if it is possible for them to integrate the new information into existing self-schemas. Finn and colleagues experimented on how to turn feedback from psychological assessment into a brief therapeutic intervention and discovered that patients found their feedback most impactful when they were first presented with test results that were close to their own understanding of themselves as persons, then with information that were somewhat discrepant from these self-schemas, and finally with the parts of information that were highly discrepant from how they thought about themselves (Finn, 1996). This led to a focus on how to engage patients from the outset of the assessment process and make the test findings relevant for them. Finn and colleagues found that gathering questions from patients about what they hoped to find out about themselves at the beginning of an assessment procedure greatly enhanced the perceived purposefulness of the assessment and promoted change (Finn, 1996).

Notably, this view echoes the *sense of coherence* conceptualized by Aaron Antonovsky (Antonovsky, 1987). Patients’ readiness to accept even potentially sensitive information obtained during assessment can be strengthened if they are initially probed about in what ways they are already searching for new perspectives in understanding themselves and the world around them (Finn, 2007). The idea of engaging patients in making the assessment meaningful for them, as straightforward as it may seem, represented a clear break with the information-gathering approach that dominated (and still dominates) psychological testing. The new focus on patient relevance “led to the practice of asking clients—in the initial assessment sessions—what puzzles, questions, or quandaries they have about themselves, and then making these questions the focus of the assessment” (Finn, 2007, p. 10). In Finn’s words:

“By assisting clients in forming questions, we invite them to ‘climb up’ with us, if you will, on an ‘observation deck’ overlooking their lives where we may begin to look jointly for answers. Many clients report that they feel relief immediately after an initial assessment session simply from having translated their inner turmoil into concrete questions.” (Finn, 2007, p. 11)

Describing the many components of Finn’s TA model—the order in which tests are administered, the provision of written feedback, etc.—in detail is beyond the scope of this article. Instead, we wish to focus on some core features of TA that we believe resonate with the CFI and which could be used to enhance the CFI even further as a potentially therapeutic tool. One of these central tenets is maintaining a stance of compassion and curiosity, where assessors seek to understand rather than to judge or classify. Here, Finn stresses that TA can indeed be used in order to diagnose patients if this is considered helpful, but that it is usually not a primary goal in the setting in which he is applying the model (Finn, Fischer, and Handler, 2012). Another central value in TA is respect for patients, as manifested in the explicit focus on asking patients what they themselves wish to find out, recognizing that patients are “experts on themselves” (Finn et al., 2012, p. 11), and inviting them to make sense of their test results. This, in turn, resembles the CFI approach by which patients are seen as authorities on their lived cultural milieux. Moreover, a core feature of TA is the desire to communicate the assessment results to the patients in a way that may be helpful for them as part of a self-transformative process, in contrast to simply providing useful information for other clinicians or decision makers (Finn, 2007). Here, test results are not viewed primarily as indicative of some objective “Truth” about patients but rather as “empathy magnifiers” (Finn, 2007, p. 38) that help clinicians get in their patients’ shoes and better understand their view of themselves and the world they inhabit.

This also means that in TA, test data are typically seen not as findings but as tools (Engelman and Allyn, 2012). The TA approach includes making use of emerging test data during the assessment process in order to inform further probing of the patient’s cognitive schemas, rather that saving them for last as if they were somehow produced by the skilled assessor and then gifted to the patient. Importantly, “[t]he goal of therapeutic assessment is not just the collection of information *about* the patient/client, but rather, the assessment procedure itself is designed to be transformative” (Engelman and Allyn, 2012, p. 71); i.e., to induce change in the way that the patient conceptualizes and copes with the issues that are subject to assessment. Moreover, in contrast to much traditional psychological assessment, the patient’s questions are viewed as more important than those of the assessor.

TA has been shown to be an acceptable and viable option for patients with personality disorders (De Saeger et al., 2014; Durosini and Aschieri, 2021), self-injurious behaviors (Ougrin et al., 2013), and eating disorders (Michel, 2002; Peters, 2000). The model has also been successfully used in work with children and adolescents (Austin, Krumholz, and Tharinger, 2012; Tharinger et al., 2009) as well as with their families (Smith et al., 2009; Tharinger et al., 2012). Moreover, it has been highlighted as a potential option in the treatment of early psychosis (Norman and Breitborde, 2014), complex traumatization (Durosini, Tarocchi, and Aschieri, 2017; Tarocchi et al., 2013), or narcissistic personality disorder (Hinrichs, 2016). While TA certainly does not provide a quick fix or a catch-all solution, it clearly emphasizes a person-centered perspective on assessment and change where patients’ conceptions of what constitutes a useful understanding in their specific context is allowed to take center stage.

## How to Use the Cultural Formulation Interview in a Therapeutic Assessment Framework

The CFI is still a work in progress informed by a research agenda (Kirmayer, 2015). As noted above, research on how the CFI can make a difference in terms of clinical outcomes on a broader scale is much needed. This includes research topics such as how use of the CFI may result in a change in diagnosis, improved treatment adherence, and a strengthened clinician–patient relationship—all of which should promote optimized treatment outcomes as well as the identification of relevant psychosocial problems “beyond diagnosis” (Kirmayer, 2015, p. 280). In addition to this, we suggest that research on how the CFI procedure (in its current version or in an adapted form) may affect clinical outcomes could prove to be a worthwhile area of study. We hypothesize that with some modifications—small scale or more comprehensive—the CFI could incorporate a TA perspective that aids in focusing the interview on the very queries that the patients have about themselves and, by extension, aim to promote change in and by itself.

Clearly, there are differences between the theoretical approaches behind TA and the CFI. Perhaps most fundamentally, the CFI is a specific instrument (albeit with a broader scope than many other such tools) intended to support culturally informed psychiatric assessment, including formal nosological categorization that is not necessarily an integral part of psychological assessment, psychotherapy, or counseling. In contrast, TA constitutes an overall attitude towards the assessment procedure. Even so, the CFI and TA share many common grounds. Certainly, a focus on patients’ subjective concerns and queries is not new to the field of cultural consultation—for example, the clinicians behind the Cultural Consultation Service in Montreal, Canada, stress the importance of discussing the patient’s own framing of vital questions before the assessment, since it may often differ substantially from the issues described in the referral (Kirmayer Jarvis and Guzder, 2014). Filippo Aschieri at the European Center for Therapeutic Assessment in Milan, Italy, describes TA as “a piano duet for four hands” (Aschieri, 2011, p. 356) in which the clinicians participate as experts in psychological theories while patients are enlisted as experts on their own lives. In this process, three levels of meaning making are integrated: the test data, the patients’ personal understanding of how these data relate to their everyday lives, and the interaction between patient and clinician. In our view, this could very well serve as a description of the ethnographic philosophy behind the CFI, whereby clinicians seek to arrive at an emic understanding. It can be noted that the CFI is, at least implicitly, informed by a second-person approach that emphasizes intersubjectivity and recognizes the alterity inherent to any encounter with “the Other” (Kirmayer, 2008, 2013; Moore and Barresi, 2017; Stanghellini, 2007). This approach presupposes difference rather than mere analogy between the world of the clinician and that of the patient—a difference that cannot fully be overcome. Rather than resignation, however, these limits of empathy call for greater efforts in jointly exploring and co-constructing meaningful narratives (Kirmayer, 2008, 2013; Stanghellini, 2007). Similarly, TA abandons a standard detached third-person assessment approach in favor of dyadic exploration where assessment findings become meaningful only in relation to the patient’s contextual lived experiences. Beyond simply noticing that the CFI and TA are both built on principles of patient centeredness, we suggest that their most salient common feature is this second-person outlook on assessment. Surely, however, this is not to say that a third-person approach (or other approaches) to the CFI can never be useful. For example, a recent implementation study suggests that first- and third-person perspectives can be combined in utilizing the CFI for psychiatric assessment and diagnostic categorization (Idar Wallin et al., 2020). Here, while the CFI was used by the staff as a more traditional third-person perspective tool in identifying “diagnostic clues,” a first-person perspective—i.e., a narrative “inside” view—still emerged in the documentation of the interview data in the patient records, where patient quotes provided “an echo of the patient’s voice” (Idar Wallin et al., 2020, p. 550). These different approaches are not mutually exclusive; the interviewer may not even be explicitly aware of them. Similarly, patients may experience therapeutic benefit from assessments that *also* serve to gather information to inform diagnosis and further clinical management (Finn and Tonsager, 1997). Nevertheless, we wish to highlight the focus on the dyadic nature of assessment as common to TA and the CFI.

This is also reflected in a similar approach to illness narratives and language. Finn has emphasized that in TA, assessors listen closely to their clients and “adopt their metaphors” (Finn, 2011, p. 25). This stance resembles the focus throughout the CFI on attending to patients’ *idioms of distress*—i.e., culturally based variations in how distress and illness are understood and expressed (Nichter, 1981)—that is made explicit in the CFI questions about how the patient frames her/his problem for members of their social network. This focus on the patient’s cultural illness prototypes and explanations becomes even more pronounced, of course, in the first CFI supplementary module that probes explanatory models in more depth. The firm TA emphasis on understanding assessment findings in the patient’s real-world context requires similar exploration and attention to idioms that are meaningful and valid in the patient’s cultural context.

There may of course be barriers that hinder the integration of a TA approach in using the CFI. For example, a potential objection is that TA may at first glance appear to require that patients have strong self-reflecting and narrativizing capabilities and that they can readily assume a position of “psychological mindedness.” However, it is not at all certain that this is necessary in order for TA to be a viable approach—in contrast, the specific emphasis on the questions and problems that the patient sees as most pressing makes TA functional regardless of whether the outlook of the patient is individualistic, sociocentric, cosmocentric, etc. Moreover, the use of TA in the treatment of early psychosis and in patients undergoing involuntary treatment (Norman and Breitborde, 2014; Toivakka, 2012) shows that it can be successfully applied in situations characterized by impaired reality testing and limited patient capacity for autonomous decision making.

A related potential barrier is the observation that many patients seen in a community setting may expect and/or prefer a more directive communication style focused on expert suggestions and explicit encouragement (Wint, 2014). Indeed, Finn cautions against a similar tendency in some patients to see the assessor as an idealized “oracle” that will offer them a definitive solution to their problems at the end of the assessment process (Finn, 2007, p. 188). Case reports from the WestCoast Children’s Clinic in Oakland, California (described briefly below), underscore that TA can be successfully applied in working with minority groups in disenfranchised communities (Guerrero, Lipkind, and Rosenberg, 2011; Rosenberg, Almeida and Macdonald, 2012). Here, interestingly, the authors note that in their experience, there is an “inherent challenge between the Eurocentric ‘task’ orientation of psychological assessment and the ‘relationship’ orientation more commonly valued within the African American community” (Rosenberg et al., 2012, p. 223), which would suggest that a dyadic, non-directive TA approach could very well be more intuitively acceptable for some individuals belonging to minority groups. Even so, it should be noted that the TA model certainly allows for clinicians to adapt their style and occasionally assume more of a guiding “expert” position when the patient needs it (Aschieri, 2016; Finn and Tonsager, 1997).

Importantly, we do not wish to stir up false promises regarding the transformative potential of TA. Even though the view of assessment as change inducing is emphasized from the outset of the TA process, it is unlikely that any one-off CFI assessment episode will lead to substantial therapeutic change. As mentioned above, TA involves a number of carefully designed subsequent techniques for eliciting further change based on the information gathered during the initial assessment. We are not suggesting that adopting a TA approach in using the CFI will somehow automatically improve treatment outcomes—what we are proposing is that explicitly engaging patients in making the interview meaningful for them can become a stepping stone in changing how they conceptualize and cope with the very issues that are subject to assessment.

What adaptations of the OCF and the CFI may be needed in order to integrate it with a TA approach, whereby patients are invited to climb up on the observation deck and make use of what they see? First, one might potentially argue that this perspective is already implicitly present in the CFI through the adoption of a second-person approach as discussed above. Even so, by explicitly addressing the importance of identifying the questions that patients’ themselves hope to answer and allowing them to take center stage in the assessment process, this perspective could be further enhanced. Below, we suggest a number of hypothetical adaptations of the content of the CFI items (i.e., the right-hand column of the instrument), the guide to the clinician performing the CFI (i.e., the instructions that precede the core CFI and the left-hand column of the instrument), and the OCF that could help incorporate a TA approach in the assessment of an individual’s social and cultural context and history. These suggestions are to be seen as illustrations of how the CFI could be modified, not as definitive edits—we readily realize that there are numerous considerations involved in updating a guideline such as the DSM section on cultural formulation. For example, as an alternative to the adaptations to the core CFI suggested below, a TA perspective could be introduced by the creation of a complementary TA-focused version of the CFI, analogous to the current supplementary modules. In this way, a TA road map would be made available without compromising the practical necessity of keeping the CFI concise (Kirmayer, 2015).

### Adaptations of the Content of Cultural Formulation Interview Items

First, it can be noted that the CFI questions are not necessarily meant to be repeated verbatim during the patient interview: “Questions may be rephrased as needed. The CFI is intended as a guide to cultural assessment and should be used flexibly to maintain a natural flow of the interview and rapport with the individual.” (American Psychiatric Association, 2013, p. 751) This means that for those clinicians who find it suitable to adopt a TA approach in performing the CFI, it is fully possible to use the instrument in its current form and adjust the questions as they go along. Still, explicitly including suggestions for how to make the interview items reflect the centrality of the patients’ queries might strengthen this perspective.

In the introductory phrase directed to the individual undergoing the interview, it is clear that although the clinician wants to know about the patient’s experience and ideas, they are first and foremost vehicles for the clinician’s own understanding: “I would like to understand the problems that bring you here so that I can help you more effectively. I want to know about your experience and ideas.” (American Psychiatric Association, 2013, p. 752) As an alternative, after the CFI introductory phrase, one might simply add an invitation for the patient to state what they themselves wish to find out and describe that the aim is to allow the interview findings to aid in this process. For example, a phrase such as “*I would like to work with you to explore what questions you have about your current situation*” could be helpful in order to cast a TA light on the CFI that shifts the perspective slightly and emphasizes the importance of the patient’s own queries about their situation. (Henceforth, all quotes in italics are our suggested adaptations of the CFI.)

Another way of integrating a TA approach to the CFI is to adapt specific interview items so as to highlight the direct importance of how the patient can make use of the findings. Perhaps the most obvious starting point is question 3, “What troubles you most about your problem?,” which could easily be adjusted so as to shift the focus to the patients’ own queries (e.g., “What troubles you most about your problem? *What would you need to find out in order to be able to change it?*”). This perspective could be further emphasized throughout the interview, such as in question 14: “What kinds of help do you think would be most useful to you at this time for your [PROBLEM]? *What would be useful for you to find out about your [PROBLEM] in order to better deal with it?*”.

Similarly, the section concerning cultural identity—a concept which is often described as somewhat difficult to grasp by patients as well as by clinicians (Aggarwal et al., 2013)—could be modified so that is explicitly stated that a purpose of the questions is to aid the patient’s own understanding of what aspects of their cultural identity that are most salient and how their cultural identity affects them in relation to the problems that they see as most important. For example, question 9 could be adjusted so as to underscore a TA perspective: “Are there any aspects of your background or identity that make a difference to your [PROBLEM]? *What aspects of your background or identity would you need to explore more in order to be able to understand and improve your situation?*” In this way, the clinician would not primarily inhabit an ethnographic stance in relation to the patient but instead assume more of a clinical co-pilot role in exploring the ways in which an individual’s own understanding of their cultural identity can become helpful and, if possible, initiate a transformative process.

### Adaptations of the Guide to the Clinician Performing the Cultural Formulation Interview

The overall approach of more clearly allowing the patient’s own queries to guide the assessment process may be further strengthened if it is explicitly mentioned as an available option in the instructions to the clinician performing the interview. For example, adjacent to the section that recommends tailoring the CFI to the individual patient context (i.e., “The CFI is best used in conjunction with demographic information obtained prior to the interview in order to tailor the CFI questions to address the individual’s background and current situation” (American Psychiatric Association, 2013, p. 751), a similar suggestion could be made regarding a TA approach: “*Moreover, ascertaining the specific questions and concerns that the individual has regarding the interplay between culture, distress, and illness at the outset of the interview and making them the focus of the assessment can increase the direct usefulness of the findings and contribute to therapeutic change.*” Likewise, the section outlining the intentions behind the CFI (i.e., “Both the person-centered process of conducting the CFI and the information it elicits are intended to enhance the cultural validity of diagnostic assessment, facilitate treatment planning, and promote the individual’s engagement and satisfaction” (American Psychiatric Association, 2013, p. 751) could be edited so as to also explicitly incorporate a potentially transformative intention.

The guidance offered in the left-hand column of the CFI could also be adapted according to a TA framework. For instance, the instruction associated with question 3, “Focus on the aspects of the problem that matter most to the individual,” may already seem to introduce a perspective similar to that of the TA approach. In its current context, however, this is probably most often taken as a recommendation to use the most urgent or relevant aspect as an example when asking about cultural perceptions and meanings; this is also reflected in the recurrent reference to this as the “[PROBLEM]” to be inserted in the succeeding CFI questions. If slightly modified (e.g., “Focus on the aspects of the problem that matter most to the individual *and how the information obtained could be useful in resolving these issues*”), this instruction could strengthen the emphasis on making the assessment meaningful for the patient. A similar perspective could be promoted throughout the CFI instructions, such as in the section regarding cultural factors affecting current help seeking where it is suggested that the clinician “[c]larify individual’s current perceived needs and expectations of help, broadly defined”. Here too, an added sentence (e.g., “*Emphasize that these perceived needs and expectations will be the focus of the assessment procedure*”) could help underscore that the patient’s queries are given full priority.

### Adaptations of the Outline for Cultural Formulation

The original DSM-IV OCF was revised for the DSM-5 and is included as an introductory framework for the CFI, highlighting the different categories of information that should be systematically assessed. As such, it might not be an obvious candidate for adaptation—the aim of adopting a TA approach in using the CFI is, after all, not to alter the *type* of information that is gathered but to further emphasize the person-centered *perspective* of the interview procedure and the way in which the findings can be put to use for the individual patient. This, however, means that the final segment of the OCF—i.e., the overall cultural assessment, in which the implications of the cultural formulation are to be summarized—could be further clarified. In fact, there is little discussion in the DSM-5 and the associated literature of how feedback to the patient about the findings generated through the use of the CFI should best be provided. In TA, as described above, there is a usually a great emphasis on the assessor’s written feedback to their patient, outlining their interpretation of the assessment findings in relation to the initial questions posed by the patient. Here, there is certainly also room for innovation of how to provide meaningful feedback to patients regarding the information collected with the CFI. Based on findings from CFI field trials in Pune, India, suggestions have been made for a further revised version of the OCF which would include, for example, summative debriefing questions about the value of the content elicited and its relevance for diagnosis and treatment planning (Paralikar et al., 2020). Such a revision could easily incorporate advice on how to offer the patient feedback on the assessment findings in a purposeful way.

## The Other Way Around: Culturally Informed Use of Therapeutic Assessment

A related question is if and how the CFI and, in a broader sense, the OCF could also further the understanding of what patient centeredness can amount to within a TA context. Although there is a paucity of research on the use of TA in community mental health settings and in culturally and racially diverse populations (Guerrero et al., 2011), a number of case reports on a culturally responsive use of TA have been published. This includes an interesting Italian clinical vignette on the use of TA in working through “microcultural differences” between a Sicilian client and a Lombardian counselor (Fantini, 2016) as well as series of case reports from the WestCoast Children’s Clinic in Oakland, California, that describe the use of TA with clients belonging to a racial minority (Guerrero et al., 2011; Rosenberg et al., 2012). Here, the use of TA to organize the assessment around “real-life” problems experienced by the clients instead of falling back on a majority-population clinician interpretation of what constitutes meaningful change was one way of overcoming initial barriers such as mistrust and disengagement.

In essence, TA can represent a break with the idea of “culture-free” assessment instruments that psychologists and psychiatrists often strive for (Smith, 2016a, b); i.e., instruments that are somehow seen as describing core features of some psychological condition regardless of the respondent’s cultural group. Assessing culturally diverse patients with the use of instruments that compare them to normative population data risks eliciting shame in the patient, not least if s/he comes to the assessment with the expectation that s/he will be misunderstood or misinterpreted (Aschieri, 2016). In contrast, the TA process aims at making assessment instruments meaningful in the patient’s specific context—“[w]ith culturally diverse clients, this means that assessors constantly ask clients to help them understand their background and traditions, so that culturally situated behaviors and attitudes are not misunderstood or pathologized” (Smith, 2016a, b, p. 4). Finn summarizes the findings in the WestCoast case reports mentioned above:

“Not only *can* Therapeutic Assessment be practiced in such settings, but I also believe that this set of articles demonstrates how perfect the model is for many underprivileged, multicultural clients. The techniques of Therapeutic Assessment are directly derived from its core values: collaboration, respect, humility, compassion, and openness/curiosity. Some clients are deeply impacted by being treated in accord with such values, and this experience in itself can be deeply healing.” (Finn, 2011, p. 23)

Owing to its background in the field of clinical psychology, much of the literature on TA revolves around the use of specific psychometric assessment instruments such as the second revision of the Minnesota Multiphasic Personality Inventory (known as the MMPI-2) or projective personality tests such as the Rorschach test. This, however, seems to be mostly tied to tradition—there is nothing that formally prevents the application of the central TA principles of enlisting patients’ curiosity, motivation, and willingness to explore their problems in the use of other instruments or techniques. Indeed, assessor flexibility in modifying test practices when it is seen as helpful for the patient and using their creativity in collaboratively applying psychological assessment instruments are explicitly mentioned as strengths of the TA model (Finn et al., 2012).

Given the numerous conceptual similarities between TA and the CFI outlined above, a straightforward strategy for strengthening a culturally responsive use of TA could be to promote the uptake of the CFI as a routine instrument within the TA community. Based on published TA case reports that focus on issues related to cultural identity, it is our belief that the CFI can prove highly useful in this context. For example, Martin and Jacklin describe working with a 27-year-old man who had migrated to the United States with his family from a Middle Eastern country as a child and who had now been referred for a learning disability assessment (Martin and Jacklin, 2012). Here, the patient’s own queries concerned his difficulties related to learning but also issues related to how growing up “in two worlds” with mixed cultural influences may create tensions within a family system. Another case report involves a 14-year-old girl who had been adopted to the United States from an Eastern European country at age 6 and who was now referred because of difficulties at school, disruptive behaviors, and self-harm (Frackowiak, 2012). Here, the assessor used TA to uncover a history of trauma and abandonment in a context of navigating the culture of “in-betweenness” associated with the girl’s status as international adoptee. In both of these cases, the CFI—and the CFI supplementary modules focused on cultural identity, school-aged children and adolescents, and immigrants and refugees in particular—could have provided helpful opportunities for discussing these issues in depth as a part of routine assessment. Similarly, in the case mentioned above involving fine-grained cultural differences between a Sicilian client and a Lombardian counselor, mainly concerning southern Italian emphasis on such concepts as familism and honor that are less salient in northern Italy (Fantini, 2016), the CFI could potentially have aided the counselor in elucidating their dissimilar points of reference.

## Conclusion

In sum, we have argued that the CFI could benefit from the application of a TA perspective by engaging patients in making the assessment meaningful for them, inducing change in how they conceptualize and cope with the issues that are subject to assessment, and avoiding treatment disruption. While there are certainly conceptual differences between the CFI and the TA approach, the models also share a number of important similarities and intersections that call for further development. Various modifications of the CFI content may be necessary in order to ensure a strengthened TA-inspired focus on what the patients wish to find out about themselves and on how to render the findings meaningful in their everyday lives. Alternatively, it may suffice to simply use the existing CFI within an explicit TA framework, much as a TA clinician would do with any assessment instrument. Moreover, we would like to highlight the CFI as a key assessment tool and promote its use among clinicians, therapists, and counselors already involved in TA. Embracing the CFI within a TA practitioner context could lead to further cross-fertilization between fields and potentially give rise to new insights into how the CFI can be optimally adapted to incorporate a therapeutic intention. We do not attempt to present a finished, ready-to-use solution for how a TA perspective could be integrated into the CFI and vice versa—instead, we would like to encourage a discussion regarding the direct therapeutic potential of the CFI and how a greater emphasis on what the patient sees as meaningful can be achieved in the use of this important assessment instrument.
